# Tensin4 is up-regulated by EGF-induced ERK1/2 activity and promotes cell proliferation and migration in hepatocellular carcinoma

**DOI:** 10.18632/oncotarget.4122

**Published:** 2015-05-12

**Authors:** Lo-Kong Chan, Yung-Tuen Chiu, Karen Man-Fong Sze, Irene Oi-Lin Ng

**Affiliations:** ^1^ Department of Pathology and State Key Laboratory for Liver Research, The University of Hong Kong, Hong Kong

**Keywords:** cten, EGF, ERK1/2 activity, HCC

## Abstract

The focal adhesion protein Tensin4, also known as cten (c-terminal tensin like), is structurally distinct from the three other members in the Tensin family. Its expression and potential functions in cancers including hepatocellular carcinoma (HCC) are not well understood. With immunohistochemistry, 43% (13/30) of our human HCC cases showed up-regulation of Tensin4 as compared with their corresponding non-tumorous livers. In HCC cells, treatment with epidermal growth factor (EGF) significantly induced Tensin4 transcript and protein expression, while treatment with pharmacological inhibitors against the MEK1/2 kinases abolished such induction, suggesting that Tensin4 expression was dependent on Ras/MAPK signaling. With immunofluorescence microscopy, the focal adhesion localization of Tensin4 was confirmed in HCC cells. Significantly, detailed examination using a panel of Tensin4 deletion constructs revealed that this specific focal adhesion localization required the N-terminal region together with the C-terminal SH2 domain. Up-regulation of ERK signaling by EGF in the HCC cells resulted in a change to a mesenchymal cell-like morphology through modulation of the actin cytoskeleton. Functionally, stable Tensin4 knockdown in SMMC-7721 HCC cells resulted in reduced cell proliferation and migration in vitro. Taken together, our data suggest that Tensin4 may play a pro-oncogenic role in HCC, possibly functioning as a downstream effector of Ras/MAPK signaling.

## INTRODUCTION

Focal adhesions are structural links between the extracellular matrix (ECM) and actin cytoskeleton [1]. Focal adhesions are composed of diverse molecules, for instance, receptors, structural proteins, adaptors, GTPase, kinases and phosphatases. These focal adhesion proteins play critical roles in normal physiological events such as cellular adhesion, movement, cytoskeletal structure and intracellular signaling pathways. In cancers, aberrant expression and altered functions of focal adhesion proteins contribute to adverse tumor behavior [2-6]. We have previously shown that focal adhesion proteins play critical roles in hepatocellular carcinoma (HCC) [7-9]. Understanding the molecular interactions and mechanisms of the interconnected focal adhesion proteins is of particular importance in understanding the mechanisms underlying HCC progression and development of potential effective treatment.

The tensin family comprises four proteins, tensin-1, -2, -3, and -4 (also known as cten), which are involved in cell migration and are localized to focal adhesion sites [1]. All four tensin family members contain the Src homology 2 (SH2) and phosphotyrosine binding (PTB) domains at the C-terminus. Through the PTB domain, tensins bind to the cytoplasmic tail of beta integrins which are transmembrane cell-adhesion molecules [10]. Growing evidence suggests that tensin proteins are not just structural proteins but also act as an important link between the ECM, actin cytoskeleton and signal transduction. Although independent studies have reported the downregulation of tensin members in various human cancer cell lines and tissues [9, 11-13], yet the mechanism underlying the role of tensins in cancers is far from clear. Tensin4 has drawn special attention in recent years due to its conserved SH2 and PTB domains at its c-terminus but lacks the N-terminus actin binding domain, making it structurally distinct from other tensins [1].

Tensin4 has been suggested to have oncogenic functions in some cancers and tumor-suppressor functions in others. It was shown to interact with RhoGAP DLC1 (Deleted in Liver Cancer 1) via its SH2 domain through an atypical, phospho-tyrosine-independent manner and strongly regulates the localization and tumor suppressive action of DLC1 [14]. In contrast, there is growing evidence supporting that Tensin4 promotes cell migration whereas knocking down of Tensin4 reduces cell migration, implicating its potential role in cancer metastasis [15-17]. Tensin4 stimulates cell motility through displacement of tensin3 at focal adhesions and consequent dissociation between the cytoplasmic tails of integrin molecules and actin fibers in a very specific MCF10A breast line [17]. Furthermore, several groups have found it to be overexpressed in breast [17, 18], gastric [19], colonic [15, 16] cancers, but under-expressed in prostatic [20] and renal carcinoma [12]. However, the expression and functions of Tensin4 in HCC have not been reported. In this study, we explored the expression, its regulation and the potential functional role of Tensin4 in HCC cells.

## RESULTS

### Expression of Tensin4 in HCC cells and human HCCs

**Figure 1:**
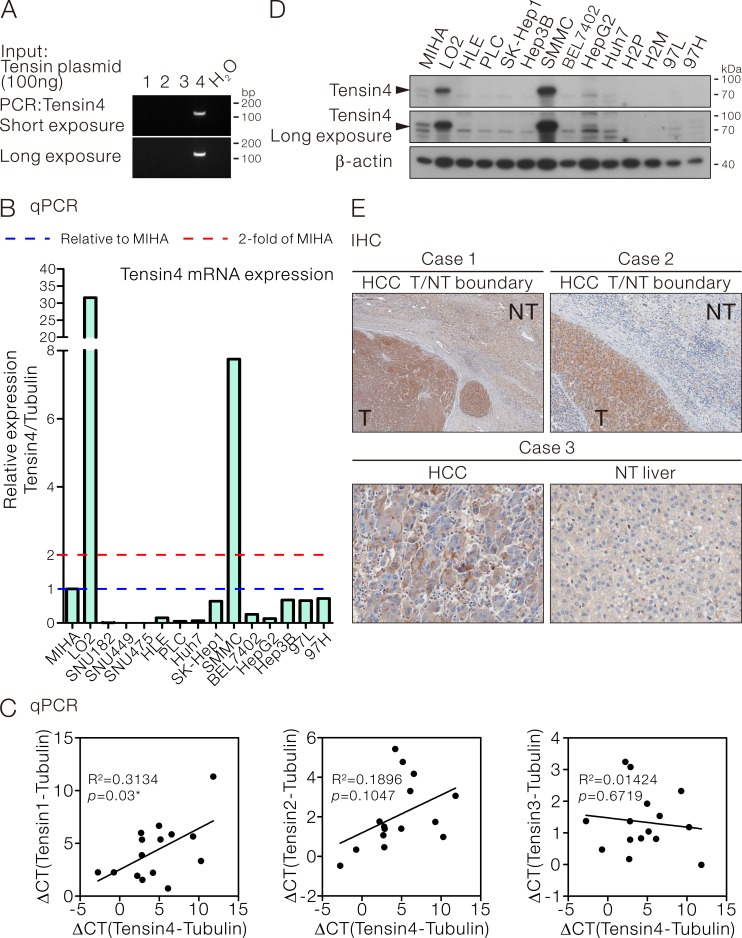
Tensin4 expression in HCC cells **A.** Specificity of the Tensin4 specific primers for qPCR assay. **B.** qPCR assay for Tensin4 transcript expression in HCC cell lines. Tubulin was served as the internal control. The normalized Tensin4 expression in each cell was displayed and compared with the immortalized liver cells MIHA. **C.** The mRNA expression levels of Tensin1, Tensin2 and Tensin3 in HCC cells in (B) were determined and subjected to regression analysis for their correlation with Tensin4 mRNA expression. The R^2^ and *p* values of their expression correlation were shown. **D.** Western blotting for Tensin4 expression in HCC cell lines. β-actin served as the normalization control. **E.** Immunohistochemistry showing overexpression of Tensin4 in three representative HCCs as compared with the corresponding non-tumorous (NT) livers.

To examine the expression of Tensin4 in HCC cells, primer pairs specifically amplifying Tensin4 but not the other Tensin family members (Tensin1-3) were employed for qPCR assay (Figure 1A). The Tensin4 expression was examined in a panel of HCC cell lines. The relative Tensin4 expression was normalized to the internal control tubulin and the normalized expression level was then compared with the immortalized normal liver cells MIHA [22] (Figure 1B). High level of Tensin4 expression was observed in LO2 and SMMC-7721 cells, while similar expression level was observed in a subset of HCC cells (SK-Hep1, Hep3B, 97L and 97H) when compared to MIHA. The Tensin4 expression was low in the remaining HCC cells and marginally detectable in the SNU-series of HCC cells tested. To have a better understanding on how Tensin4 transcript expression correlated with that of the other Tensin members, Tensin1, -2 and -3 expression were detected in HCC cells with specific qPCR assays in the same panel of HCC cells (Supplementary Figure 1A and B). Regression analysis of the expression data among individual Tensin members with Tensin4 revealed a positive correlation between Tensin1 and Tensin4 (R^2^ = 0.3134, *p* = 0.03). No significant correlations were found among the Tensin2, -3 and -4 expression. With the use of Tensin4-specific antibodies recognizing the Tensin4 PTB domain as epitope (Supplementary Figure 2), we observed the protein expression pattern of Tensin4 was comparable to the transcript expression in HCC cells (Figure 1D). Similarly, comparable transcript and the corresponding protein expression was also observed for Tensin3 in HCC cells (Supplementary Figure 1C). With immunohistochemistry on a cohort of 30 randomly selected, surgically resected primary HCC samples from patients (Supplementary Table 1), overexpression of Tensin4 in HCCs, as compared with their corresponding non-tumorous livers, was found in 43% (13/30) of the cases (Figure 1E).

### The SH2 domain and the N-terminal region together were important for proper Tensin4 focal adhesion localization in HCC cells

**Figure 2:**
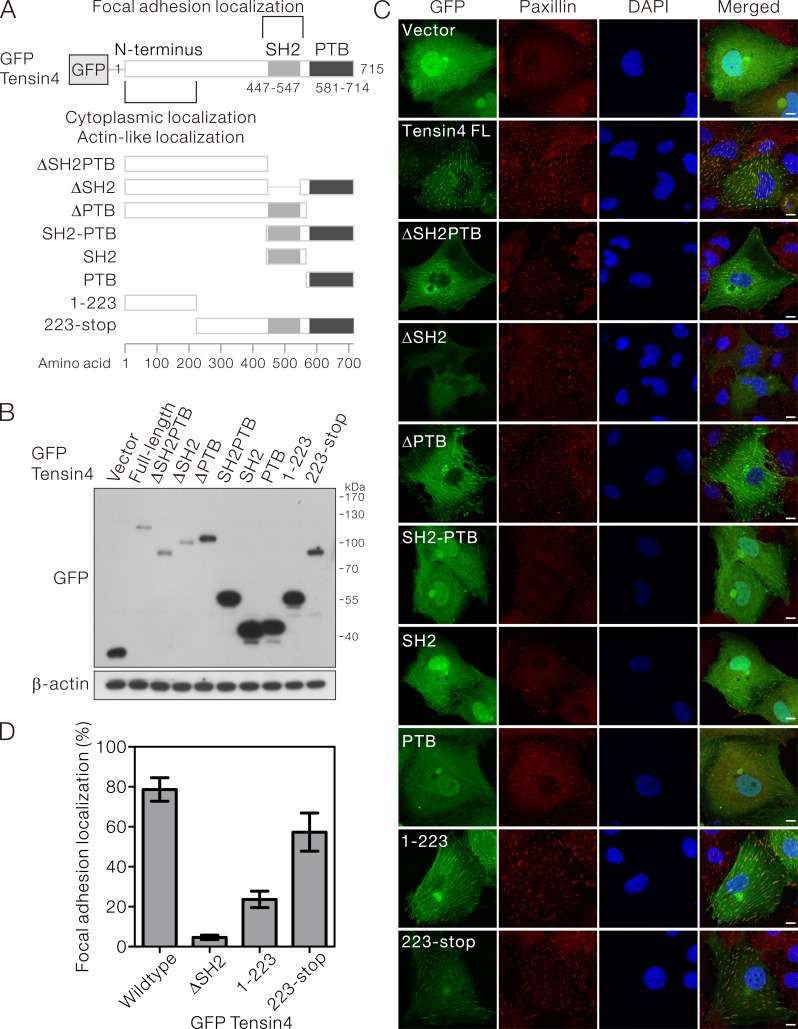
SH2 domain was required for the focal adhesion localization of Tensin4 **A.** Schematic diagram showing the structure of the N-terminal GFP-tagged Tensin4 expression constructs with specific functional domain being removed for the subsequent subcellular localization analysis. **B.** The Tensin4 constructs listed in (A) were transiently transfected in HLE cells and the cell lysates were subjected for western blotting against anti-GFP antibodies. **C.** The localization of various GFP-Tensin4 proteins was examined by confocal microscopy. The coverslips were counterstained with paxillin and DAPI for focal adhesions and cell nuclei. Scale bar: 10 μm. **D.** The percentages of the positive focal adhesion localization of a subset of GFP-Tensin4 mutant were quantified by counting as least 50 transfected HLE cells. The mean values and corresponding SDs were obtained from three independent experiments.

Although Tensin4 is structurally distinct from other Tensin family members, it possesses the characteristic focal adhesion localization. However, the contribution of individual structural domains to this subcellular localization is unclear. To answer this, we cloned a panel of expression constructs that drove the expression of N-terminal GFP-fusion Tensin4 with its functional domains being removed individually or in combination (Figure 2A). The expression constructs were then transfected into HLE cells which had low endogenous Tensin4 expression. Successful expression of the specific Tensin4 variants was confirmed by Western blotting, showing protein bands of expected molecular size (Figure 2B).

With confocal microscopy, we observed that GFP-Tensin4 showed a punctate staining in the cytoplasm perfectly co-localizing with the focal adhesion marker, paxillin. This focal adhesion localization partially required the presence of SH2 domain, as Tensin4 variants ΔSH2 and ΔSH2PTB lacking the SH2 domain were less localized to the focal adhesions. Focal adhesion localization was not affected without the PTB domain, as demonstrated by the ΔPTB mutant (Figure 2C, upper part). Expression of GFP-SH2 and -PTB alone or -SH2-PTB showed non-specific cellular staining resembling the GFP (Figure 2C, middle part). In contrast, the Tensin4 223-stop variant, which contained the central region in addition to the SH2-PTB domain, showed focal adhesion localization as well as unexpected nuclear localization. The most N-terminal Tensin4 variant 1-223 showed actin-like localization in the cytoplasm, suggesting that it played a critical role in maintaining the cytoplasmic localization of Tensin4 (Figure 2C, lower part). To further evaluate the relative importance of the N-terminal region and the C-terminal SH2 domain in contributing the focal adhesion localization of Tensin4, we quantified the focal adhesion localization pattern among the Tensin4ΔSH2, 1-223 and 223-stop with respect to the wild type control in HLE transfected cells (Figure 2D). Our data suggested that C-terminal SH2 region played a significant role in determining the focal adhesion localization of Tensin4. Besides, the N-terminal region might also play an auxiliary role in supporting this localization with the observation that N-terminal region also showed only a certain percentage of focal adhesion localization, while lacking this N-terminal region slightly reduced the degree of this localization (Figure 2D). Taken together, focal adhesion targeting Tensin4 required more than just the SH2 domain and likely involved also the N-terminal region.

### Tensin4 expression was up-regulated by EGF in a MAPK-dependent manner

**Figure 3:**
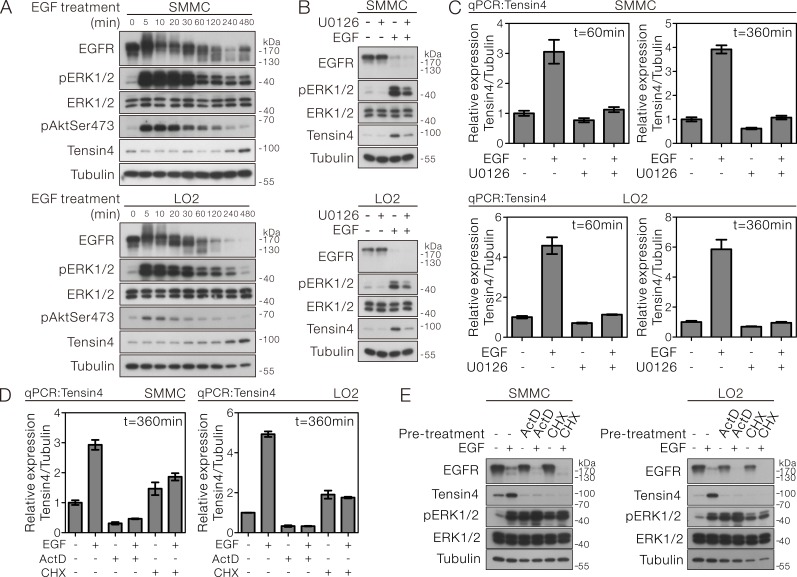
EGF-induced Tensin4 up-regulation required ERK1/2 activation followed by transcription- and translation-dependent mechanisms **A.** SMMC-7721 and LO2 cells were treated with EGF at a concentration of 40 ng/ml for the indicated duration. The cell lysates were then collected and subjected to Western blotting for the indicated proteins. **B.** SMMC-7721 and LO2 cells were pre-treated with an MEK inhibitor U0126 for 1 hour prior to EGF treatment for 6 hours. The cell lysates were then collected and subjected to Western blotting for the indicated proteins. **C.** SMMC-7721 and LO2 cells were pre-treated with MEK inhibitor U0126 for 1 hour prior to EGF treatment for 1 and 6 hours, respectively. The Tensin4 mRNA transcript level was determined by qPCR with normalization to tubulin. **D.** SMMC-7721 and LO2 cells were pre-treated with either transcriptional inhibitor Actinomycin D (ActD) or translational blocker Cycloheximide (CHX) for 1 hour prior to EGF treatment for 6 hours. The cell lysates were then collected and subjected to Western blotting. **E.** The Tensin4 mRNA transcript level was determined by qPCR with normalization to tubulin in SMMC-7721 and LO2 cells and subjected to treatment in (D).

The expression of Tensin4 in HCC cells led us to speculate the possibility of its regulation under the mitogen stimulation. We treated the SMMC-7721 and LO2 cells, which had good expression of Tensin4, with EGF, followed by detection of Tensin4 protein expression in a time course manner. Successful EGF treatment was indicated by the EGFR activation. The EGFR band shifted upward 5 minutes after stimulation and was gradually inactivated through protein down-regulation at the later time points. Importantly, the Tensin4 level was progressively up-regulated after EGF treatment and the protein expression level reached the maximum at 8 hours (Figure 3A). To understand the signaling underlying the EGF-induced Tensin4 expression in HCC cells, we examined the activation status of the two best known downstream pathways of receptor tyrosine kinase, the Ras-MAPK and PI3K-Akt signaling. Interestingly, upon EGF treatment, the phospho-ERK1/2 activity was strongly and persistently up-regulated throughout the experiment, while the total ERK1/2 levels remained unaltered. In contrast, the phospho-Akt level was only transiently up-regulated in both cells. Pre-treatment with the pharmacological MEK inhibitor U0126 in both SMMC-7721 and LO2 cells markedly diminished the EGF-induced Tensin4 protein up-regulation (Figure 3B), showing that the expression of Tensin4 was strongly dependent on the phospho-ERK1/2 activity. Pre-treatment with Rapamycin, which acts against the PI3K-Akt downstream, mTORC1, could not block the EGF-induced Tensin4 protein up-regulation (Supplementary Figure 3). We further confirmed these observations at the transcript level by showing the robust up-regulation of Tensin4 mRNA level in SMMC-7721 and LO2 cells upon EGF induction, while U0126 treatment consistently suppressed the increase in Tensin4 mRNA levels (Figure 3C). To further determine that the EGF-induced Tensin4 expression was mediated by mRNA transcription followed by protein translation, we pre-treated the SMMC-7221 and LO2 cells with either the transcriptional inhibitor Actinomycin D (ActD) or the translational blocker Cycloheximide (CHX), followed by Tensin4 mRNA and protein detection. qPCR analysis showed that both the basal and EGF-induced Tensin4 mRNA expression levels were effectively suppressed by ActD treatment, while CHX treatment led to a slight up-regulation of basal Tensin4 mRNA transcript (Figure 3D). Nevertheless, both treatments effectively blocked the EGF-induced Tensin4 protein up-regulation (Figure 3E). Collectively, these observations suggested the EGF-induced Tensin4 expression involved the *de novo* Tensin4 mRNA transcription and protein translation.

### Persistent ERK1/2 activation was required for EGF-induced Tensin4 expression

**Figure 4:**
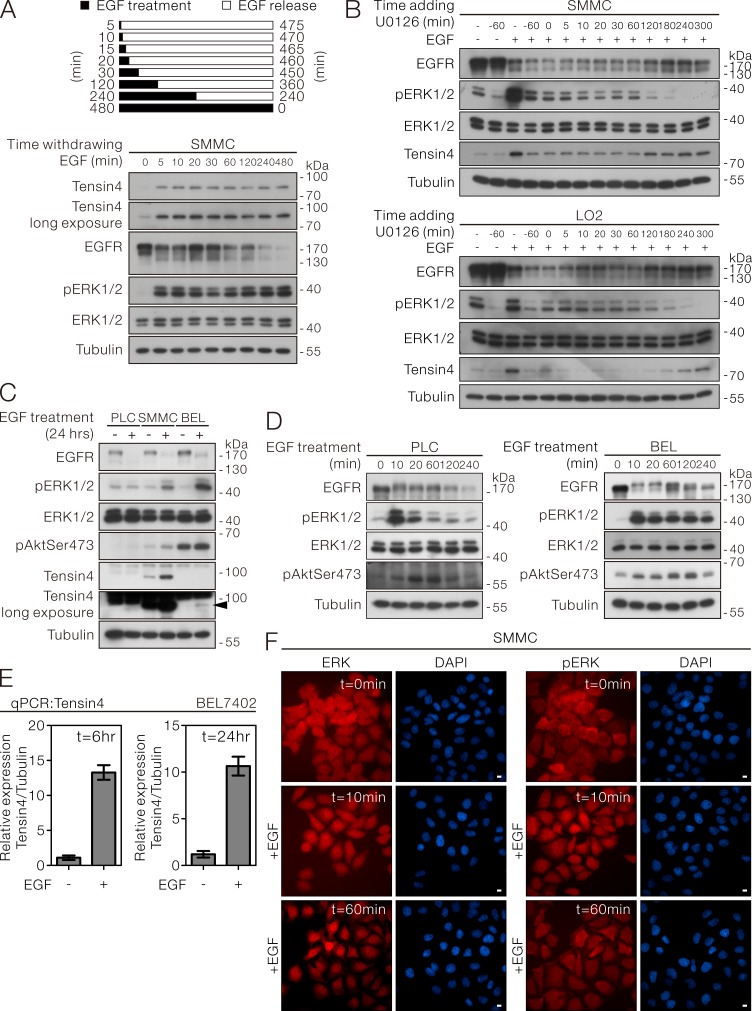
EGF-induced Tensin4 up-regulation required persistent ERK activation underlying ERK nuclear translocation **A.** SMMC-7721 cells were treated with EGF for the indicated durations and then released by washing twice with PBS followed by incubation for up to 8 hours. The cell lysates were then collected and subjected to Western blotting. **B.** SMMC-7721 and LO2 cells were induced with EGF treatment plus pre-treatment or post-treatment with the MEK inhibitor U0126 at the indicated time points. All the samples were collected at 360 minutes (6 hours) and subjected to Western blotting for the indicated proteins. **C.** PLC, SMMC-7721 and BEL7402 cells with different basal Tensin4 expression level were treated with EGF for 24 hours. The cell lysates were then collected and subjected to Western blotting. The arrowhead highlights the induction of Tensin4 in BEL7402 cells with low basal Tensin4 expression. **D.** PLC and BEL7402 cells were treated with EGF at 40ng/ml for the indicated durations. The cell lysates were then collected and subjected for Western blotting. **E.** The Tensin4 mRNA expression level in BEL7402 cells after EGF induction at 6 and 24 hours were determined by qPCR comparing with the untreated control. **F.** SMMC-7721 cells subjected to EGF induction for the indicated time points were fixed and subjected to immunofluorescence detection for ERK1/2 and phospho-ERK1/2. The coverslips were counterstained with DAPI for nuclei. Scale bar: 10 μm.

In SMMC-7721 cells, we noticed that transient EGF-treatment as short as 5 minutes, followed by incubation in the absence of EGF till the 8-hour time point, was sufficient to trigger the signaling required for up-regulating Tensin4 expression. Notably, the increased length of EGF treatment only inversely correlated with the level of EGFR but did not affect the degree of ERK activation and the final Tensin4 protein expression level (Figure 4A). This observation prompted us about the possible importance of persistent ERK1/2 in triggering the Tensin4 up-regulation. To demonstrate that persistent ERK1/2 activity was required for EGF-induced Tensin4 up-regulation, we speculated that blocking the ERK1/2 activation by U0126 at designated time points after EGF induction would diminish the Tensin4 up-regulation. Consistent to what we had shown in Figure 3B, pre-treatment of U0126 blocked the EGF-induced Tensin4 induction. Importantly, we found that addition of U0126 at early time points (ranging from 5 to 240 minutes) after EGF treatment effectively lowered the pERK1/2 activity, which was followed by the Tensin4 induction, in both SMMC-7721 and LO2 cells (Figure 4B, upper and lower panel). Interestingly, although U0126 addition at 300 minutes (60 minutes before the experimental endpoint of EGF induction) could transiently suppress pERK1/2 activity, its effect in suppressing Tensin4 induction was not obvious, probably because the requirement of the persistent ERK1/2 activation had been fulfilled in the first 300 minutes of the EGF treatment. To further examine whether EGF treatment was sufficient to upregulate Tensin4 expression in HCC cells with relatively low Tensin4 expression, PLC (very low expression) and BEL7402 (low expression) HCC cells were subjected to prolonged EGF treatment for 24 hours, with SMMC-7721 cells serving as a control. Interestingly, EGF-induced up-regulation of Tensin4 was only observed in BEL7402 but not PLC cells, possibly due to failure of increase in ERK1/2 activity in PLC cells (Figure 4C). Careful examination of the earlier time points of EGF treatment indicated that PLC cells did respond to the EGF stimulation, with the maximum ERK1/2 activity detected at 10 minutes, but it was followed by a quick reduction back to the basal level at 2 hours (Figure 4D, left panel). In contrast, the ERK1/2 activity was maintained at a high level in BEL7402 cells after EGF stimulation (Figure 4B, right panel). Consistently, the Tensin4 protein up-regulation in BEL7402 cells was coupled with the increase in the transcript level as in other cells (Figure 4E). Besides the ERK1/2 activity, spatial changes in terms of an increase in nuclear accumulation of ERK1/2 and pERK1/2 started to be observable shortly after 10 minutes of EGF stimulation in SMMC-7721 cells (Figure 4F). A strong shifting of ERK1/2 localization from cytoplasm to nucleus was observed 60 minutes after the EGF treatment. Similar observation had also been confirmed in LO2 cells (Supplementary Figure 4). The above observations suggested that EGF-induced Tensin4 up-regulation requires persistent ERK activation underlying ERK nuclear translocation.

### EGF-induced Tensin4 up-regulation was associated with morphological changes

**Figure 5:**
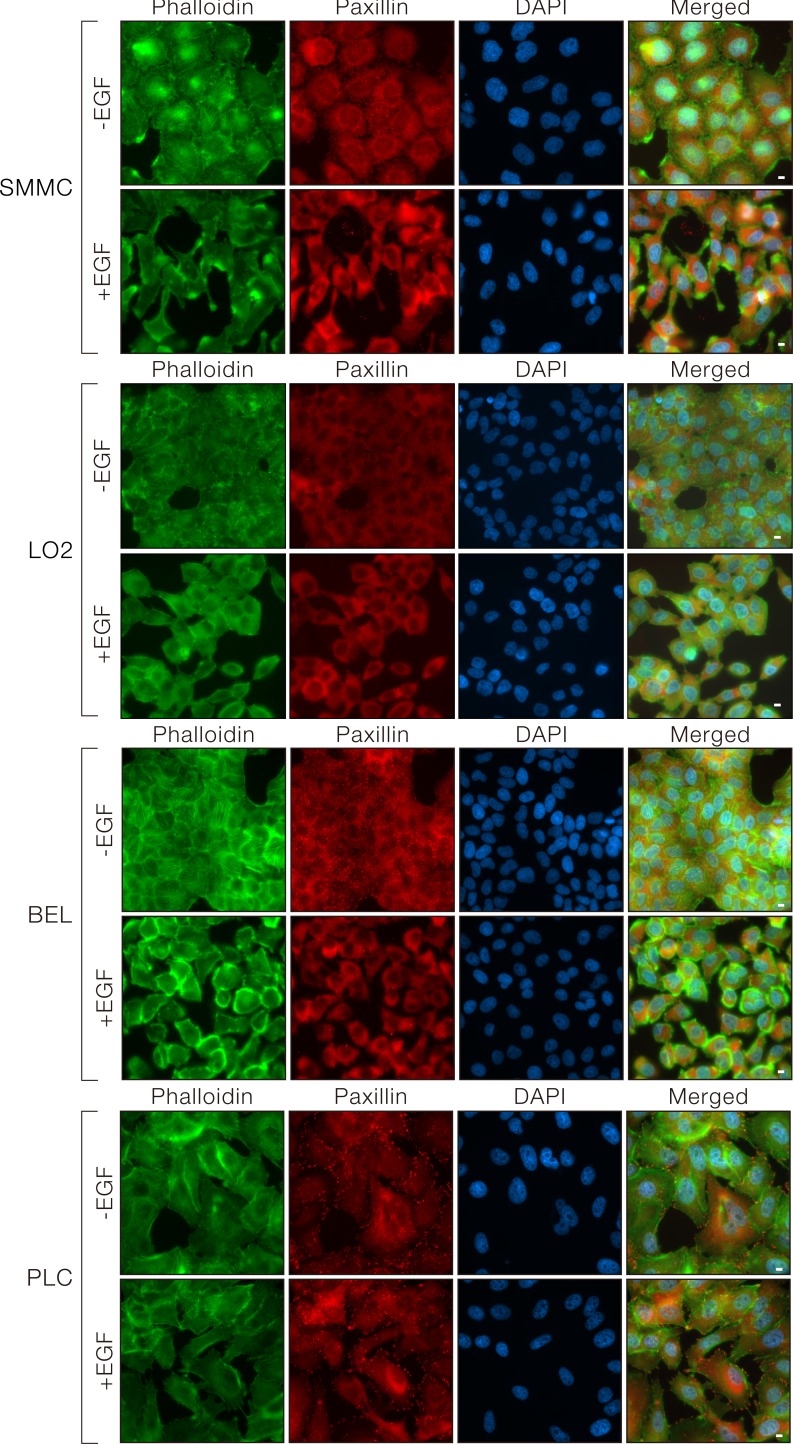
EGF induced morphological changes in cells with Tensin4 up-regulation SMMC-7721, LO2, PLC and BEL7402 cells were treated with EGF at a concentration of 40ng/ml for 24 hours. The cells were fixed and counterstained with phalloidin and DAPI for F-actin and cell nuclei. Scale bar: 10 μm.

In addition to the underlying signaling changes, we observed significant morphological changes of the SMMC-7721 and LO2 cells upon EGF treatment. SMMC-7721 and LO2 cells changed from their uniform epithelial monolayer at the basal state to a scattered organization with an elongated, mesenchymal cell-like shape. The cellular structural re-organization was visualized by the strong F-actin staining around the cell periphery and at the cell protrusions. The focal adhesions were also re-organized and became intensely stained at the actin protrusions (Figure 5, upper two panels). The dramatic cytoskeletal re-organization after EGF treatment in BEL7402 (Figure 5, lower panel) resembled the observations made in SMMC-7721 and LO2 cells. However, limited EGF-induced morphological changes were observed in PLC cells, probably due to the limited EGF-induced downstream ERK signaling with no observable Tensin4 induction (Figure 5, bottom panel). Taken together, EGF-treatment induced Tensin4 expression through up-regulating ERK1/2 signaling, with associated cell morphology changes through modulation of the actin cytoskeleton.

### Stable Tensin4 knockdown suppressed cell proliferation and EGF-induced cell migration

To assess the biological function in Tensin4 expression in HCC cells, we established two stable shTensin4 knockdown clones (sequence −16 and −24) in SMMC-7721 cells by lentiviral transduction followed by puromycin selection. Successful Tensin4 knockdown were confirmed by Western blotting and compared with the non-targeted control (NTC) cells (Figure 5A). At the basal level (without EGF treatment), stable Tensin4 knockdown did not significantly alter the expression level and activity of EGFR and ERK1/2 (Figure 5A). Tensin4 knockdown cells remained sensitive to EGF, with up-regulation of the ERK1/2 activity after EGF treatment (Figure 5A), further confirming that Tensin4 is downstream of ERK.

Since Tensin4 expression in HCC cells was tightly regulated by EGF-MAPK signaling axis, which is strongly associated with cell growth and migratory ability, we queried whether Tensin4 knockdown might also have similar effects in HCC cells in these biological activities. In the cell proliferation assay, we observed that both Tensin4 knockdown SMMC-7721 cells showed a significant reduction in cell proliferation rate (Figure 5B). In addition, these stable cells also displayed reduced morphological changes in response to EGF treatment, in accordance with the degree of Tensin4 knockdown efficiency (Figure 5C) and significant reduction in cell migration rate using the transwell assay with EGF as chemoattractant (Figure 5D). Taken together, these observations suggest that Tensin4, being a downstream target of ERK kinase, plays a significant role in promoting cell proliferation and migration in HCC cells due to Ras-ERK signaling-mediated Tensin4 up-regulation (Figure 5E).

## DISCUSSION

Tensin4 was originally identified as the smallest Tensin family member and with tissue-specific expression in prostate and placenta [13]. However, it was later found that Tensin4 was also expressed in other normal human tissues such as prostate, breast, colon, lung and stomach at different basal levels [19]. Dysregulation of expression of Tensin4 has been suggested in various forms of human cancer [13, 16, 17, 19, 20, 23-26]; however, Tensin4 expression has not yet been documented in HCC.

In this study, we found that Tensin4 was expressed in human HCC cells, though the expression level was quite variable among the different cell lines. The Tensin4 antibodies used in our study successfully detected endogenous Tensin4 protein in HCC cell lines, with an expected band size and a low non-specific background. We further used the same antibodies to performed immunohistochemistry for Tensin4 expression in human HCC tissue sections. In our cohort of 30 HCC cases, we found overexpression of Tensin4 in 43% of the tumors. In this study, we specifically focused on the mitogen expression regulation and functional role of Tesnin4 in HCC cell line model.

**Figure 6:**
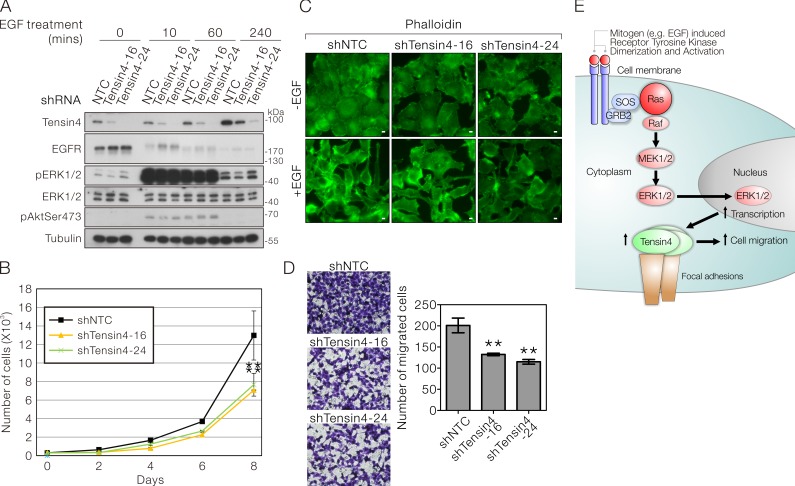
Stable Tensin4 knockdown suppressed HCC cell proliferation and EGF-induced cell migration **A.** Establishment of two independent shTensin4 (−16 and −24) stable knockdown in SMMC-7721 cells by lentiviral transduction. The cell lysate of the stable knockdown cells and the corresponding non-targeted control (NTC) cells with or without the treatment of EGF were subjected to Western blotting for Tensin4 and various signaling components in EGF-ERK signaling. **B.** shTensin4 and the control SMMC-7721 cells were subjected to cell proliferation assay for eight days to assess the growth rate. shTensin4 stable cells proliferated significantly more slowly (*p < 0.01*; *t*-test). Data were obtained from three independent experiments. **C.** shTensin4 and control SMMC-7721 cells were subjected to EGF treatment for 12 hours, followed by immunofluorescence detection for F-actin with FITC-conjugated Phalloidin. Corresponding untreated control was also include (D) Scale bar: 10 μm. **D.** shTensin4 and the control SMMC-7721 cells were subjected to EGF-induced transwell migration assay for 12 hours. shTensin4 stable cells migrated significantly more slowly (*p < 0.01*; *t*-test) Data were obtained from three independent experiments. **E.** Schematic diagram illustrating the current findings on how growth factor such as EGF up-regulates Tensin4 through Ras-MAPK signaling and its potential role in supporting cell migration.

Our current work provides a better understanding on both the molecular regulation and functional role of Tensin4 in HCC cells. Firstly, we systematically analyzed the subcellular localization of Tensin4 in HCC cells and demonstrated the requirement of SH2 domain plus the N-terminal region for its proper focal adhesion localization in cytoplasm. To our surprise, in addition to focal adhesion localization, truncation of the N-terminal region of Tensin4 was found to display a certain degree of nuclear staining. However, preliminary analysis of the primary amino acid sequence of Tensin4 did not identify any classically defined Nuclear Localization Signal (NLS). Tensin4 has been previously suggested to play tumorigenic role with β-catenin in the nucleus in colorectal cancer cells *in vitro* [20]. However, it is currently unclear how prevalent nuclear Tensin4 is and the exact mechanisms Tensin4 and β-catenin involved in driving tumorigenesis.

With the use of more HCC cell models, we defined several key requirements that are required for EGF-induced Tensin4 up-regulation, which included intact EGFR for EGF interaction, persistent ERK1/2 activation after EGF stimulation, and a reasonable amount of basal expression of Tensin4. Furthermore, functional transcription and translational machineries are required to support the Tensin4 up-regulation. We clearly demonstrated that HCC cells with the above requirements had Tensin4 induction after EGF treatment, followed by significant morphological changes through cytoskeletal remodeling. Based on our work done so far, mitogen and cytokine other than EGF including hepatocyte growth factor (HGF) and interleukin-6 (IL-6) could not induce Tensin4 expression (Supplementary Figure 5), probably due to failure of stimulating persistent ERK activity. Other study has suggested that various growth factors and cytokines such as FGF and IL6 can induce Tensin4 expression in human prostate and colon cell lines [27]. However, whether their actions are cell-type specific or not remains to be confirmed. We demonstrated an EGF-induced ERK nuclear accumulation in SMMC-7721 and LO2 cells, which may probably be associated with Tensin4 expression. However, the mode of interaction between the nuclear ERK and the transcription regulator(s) of Tensin4 remains to be explored and defined. Currently, no successful attempt has been made [28].

Using stable shTensin4 knockdown model in SMMC-7721, we demonstrated that Tensin4 was required for cell proliferation and EGF-induced cell migration in HCC cells, suggesting an oncogenic role of Tensin4 in HCC cells. The role of Tensin4 in cell proliferation has been controversial [11, 15, 16, 29]; our observation that Tensin4 affected SMMC-7721 cell proliferation upon knockdown may probably due to its high expression in SMMC-7721 cells. Although our results demonstrated the important role of Tensin4 in cell proliferation and migration, other molecular players that are potentially involved in Tensin4-associated function are not clearly understood. Previously, it has been suggested that integrin-linked kinase (ILK) may work as a downstream target of Tensin4 to mediate its oncogenic function in colorectal cancer [23]. Although we have previously suggested the role of ILK in HCC cell migration [30], we did not find a reduction in the ILK expression level after Tensin4 knockdown or an up-regulation in the ILK expression level after EGF stimulation (data not shown). The inconsistencies of the data regarding the signaling network linking up with Tensin4 functions again suggest its diverse roles in different tissue types through the utilization of different signaling pathways, and further investigation on this would be critical.

Taken together, our work has added an important piece of data to the role of Tensin4 in human HCC cells. Further functional exploration will help to address the actual molecular role of Tensin4 in mediating the biological effects in cell proliferation and migration. We look forward to the use of more relevant *in vitro* and *in vivo* models resembling the actual physiological environment to confirm the biological importance of Tensin4 in HCC and other human cancers.

## MATERIALS AND METHODS

### Plasmids

Tensin4 expression constructs using pEGFP-C1 vector (BD Biosciences Clontech, Palo Alto, CA) were prepared by standard molecular cloning techniques and PCR amplification of the described fragments using the pEGFP-C1-cten as the template (a gift provided by Dr. Y. Yarden of Weizmann Institute of Science, Israel). The GFP-tagged expression vector, pEGFP-C1 carrying Tensin4 1-446 (ΔSH2PTB), Δ447-547 (ΔSH2), 1-580 (ΔPTB), 442-715 (SH2PTB), 442-566 (SH2), 567-715 (PTB), 1-223, and 223-715 (233-stop), respectively, were constructed. All the DNA expression constructs were confirmed by DNA sequencing. shTensin4 and non-targeted control (NTC; SHC002) lenti-viral based pLK0.1-Puro constructs were purchased from Sigma (Sigma, St. Louis, MO, USA). The NTC construct carried a scrambled sequence 5′-CAACAAGATGAAGAGCACCAA-3′ set out by RNAi consortium.

### Cell culture, plasmid transfection, stable knockdown cells establishment

SMMC-7721, LO2 and BEL7402 cells were cultured in DMEM high-glucose medium supplemented with 10% fetal bovine serum, penicillin and streptomycin at 37°C with 5% CO2 in air. HLE and PLC cells were cultured in DMEM-low glucose medium and MEM medium supplemented with 10% fetal bovine serum, penicillin and streptomycin under the same conditions. For HLE cell transfection, cells were transfected with Lipofectamine 2000 reagent (Invitrogen, Carlsbad, CA) as previously described [8]. Briefly, 1μg plasmid was mixed with 2.5μl Lipofectamine 2000 in 100μl serum free DMEM medium and incubated for 20 minutes at room temperature. The mixture was then added to HLE cells seeded on 12-well format (8×10^4^cells/well) for 18 hours. For stable shRNA lenti-viral transduction, positive shTensin4 knockdown cells and shNon-Targeted Control (NTC) cells were selected with Puromycin at a concentration of 500ng/ml for 7 days after viral transduction.

### Epidermal growth factor (EGF) and inhibitor treatment

1.5×10^5^ cells were seeded overnight in 12-well format and stimulated with 40ng/ml EGF (Cell Signaling Technology, Beverly, CA) in full growth medium for the indicated duration. Working concentration of 10μM MEK inhibitor U0126 (Cell Signaling Technology), 1μg/ml transcriptional inhibitor Actinomycin D (Sigma) and 50μg/ml translational inhibitor Cycloheximide (Sigma) were applied to the cell culture in specific experiment at the indicated time point and duration accordingly. All inhibitors were dissolved in DMSO.

### Human HCC clinical samples

Human HCCs and their corresponding non-tumorous liver samples of 30 randomly selected primary HCCs were obtained from patients with liver resection between 1991 and 2001 at Queen Mary Hospital, Hong Kong. All specimens were either snap-frozen in liquid nitrogen and stored at −80°C, or fixed in 10% formalin for paraffin embedding. The use of human specimens was approved by the institutional review board of the University of Hong Kong/Hospital Authority Hong Kong West Cluster (UW09-185). Among the 30 patients, 23 were male and 7 female. The age ranged from 24-74 years (mean = 52.9 years). Twenty-four patients were serum positive for hepatitis B surface antigen (HBsAg); 6 were negative. The clinicopathological data of the patients are listed in Supplementary Table 1.

### Real-time qPCR for Tensin4 expression

Specific forward and reverse primers for Tensin1, -2, -3 and -4 detection as described by Cao et al. [21] were employed to detect Tensin1, -2, -3 and -4 expression in HCC cells with the use of SYBR Green PCR Master Mix (Applied Biosystems, Foster City, CA). Primer pairs 5′-aacacggatgagacctactgcat-3′ and 5′-gggtgcggaagcagatgt-3′ were used for tubulin detection. qPCR reaction was performed on a Applied Biosystems 7900HT Fast Real-Time PCR System. The relative comparison ΔΔCT method was used for gene expression analysis with tubulin used as internal control.

### Protein lysate preparation and western blotting

Cell culture in 12-well format was washed once with PBS and subjected to the direct SDS-lysis with 60ul standard Laemmli sample buffer with DTT. The whole cell lysate were boiled for 10 minutes. One-tenth of the cell lysate was subjected to 10% SDS-PAGE analysis. Most of the immunoblotting detection (EGFR, phospho-ERK1/2 Thr202/Tyr204, ERK1/2, phospho-Akt Ser473) was performed using antibodies from Cell Signaling Technology at a dilution of 1:1000 in 4% BSA/TBST as suggested by the manufacturer. Other proteins were detected with the use of anti-Tensin4 (ab82178) antibody (1:500, Abcam, Cambridge, MA, USA) in 4% BSA/TBST, anti-GFP antibody (1:1000, Santa Cruz Biotechnology), and anti-tubulin antibody (1:5000, Sigma) in 4% milk/TBST respectively.

### Immunofluorescence microscopy and immunohistochemistry

Cells seeded on coverslips with were fixed with 4% paraformaldehyde in PBS for 15 minutes and then permeabilized with 0.1% Triton-X-100 in PBS for 10 minutes, followed by blocking with 3% bovine serum albumin in PBS for 20 minutes at room temperature. The blocked coverslips were stained with anti-paxillin antibody (1:400, EMD Millipore, Billerica, MA), followed by AlexaFluor-594-conjugated secondary antibody (Invitrogen) to visualize focal adhesions. The F-actin and nuclei were visualized by TRITC-labeled phalloidin and DAPI (Sigma). For immunofluorescence detection for ERK1/2 (1:50, ERK1/2 antibody) and pERK1/2 (1:500, pERK1/2 antibody), methanol permeabilization (IF Methanol-perm) was performed accordingly with the protocol set out by the manufacturer (Cell Signaling Technology). The processed coverslips were mounted in Vectashield anti-fade mounting medium (Vector Laboratories, Burlingame, CA) Images were captured by a Carl Zeiss LSM700 laser scanning confocal microscope. Immunohistochemistry was performed on formalin-fixed, paraffin-embedded sections from resected livers from HCC patients, using rabbit polyclonal antibody against IHC capable anti-Tensin4 (ab82178) antibody (Abcam) at 1:50 dilution.

### Cell proliferation assay

1×10^3^ cells of Tensin4 knockdown and control cells were seeded overnight in triplicate in 24-well format and cultured for 8 days. The total number of cells were counted and recorded every two days with the use of the Z1 particle counter (Beckman Coulter, Fullerton, CA).

### Transwell migration assay

1×10^5^ SMMC-7721 cells in 100ul serum-free medium were seeded on the top chamber of the transwell and stimulated to migrate with 20ng/ml EGF added at the lower chamber for 12 hours. The migrated cells were fixed with methanol and stained with 0.04% crystal violet solution for 20 minutes. The number of migrated cells was counted and recorded accordingly.

## SUPPLEMENTARY MATERIAL FIGURES AND TABLE


