# Supplementary material for: Tensin4 is up-regulated by EGF-induced ERK1/2 activity and promotes cell proliferation and migration in hepatocellular carcinoma
Source: Oncotarget. 2015 May 12;6(25):20964–76. doi: 10.18632/oncotarget.4122 (PMC4673243; doi:10.18632/oncotarget.4122)
Supplement: Supplementary file 1 [file oncotarget-06-20964-s001.pdf]

Tensin4 is up-regulated by EGF-induced ERK1/2 activity and promotes cell proliferation and migration in hepatocellular carcinoma

Supplementary Material

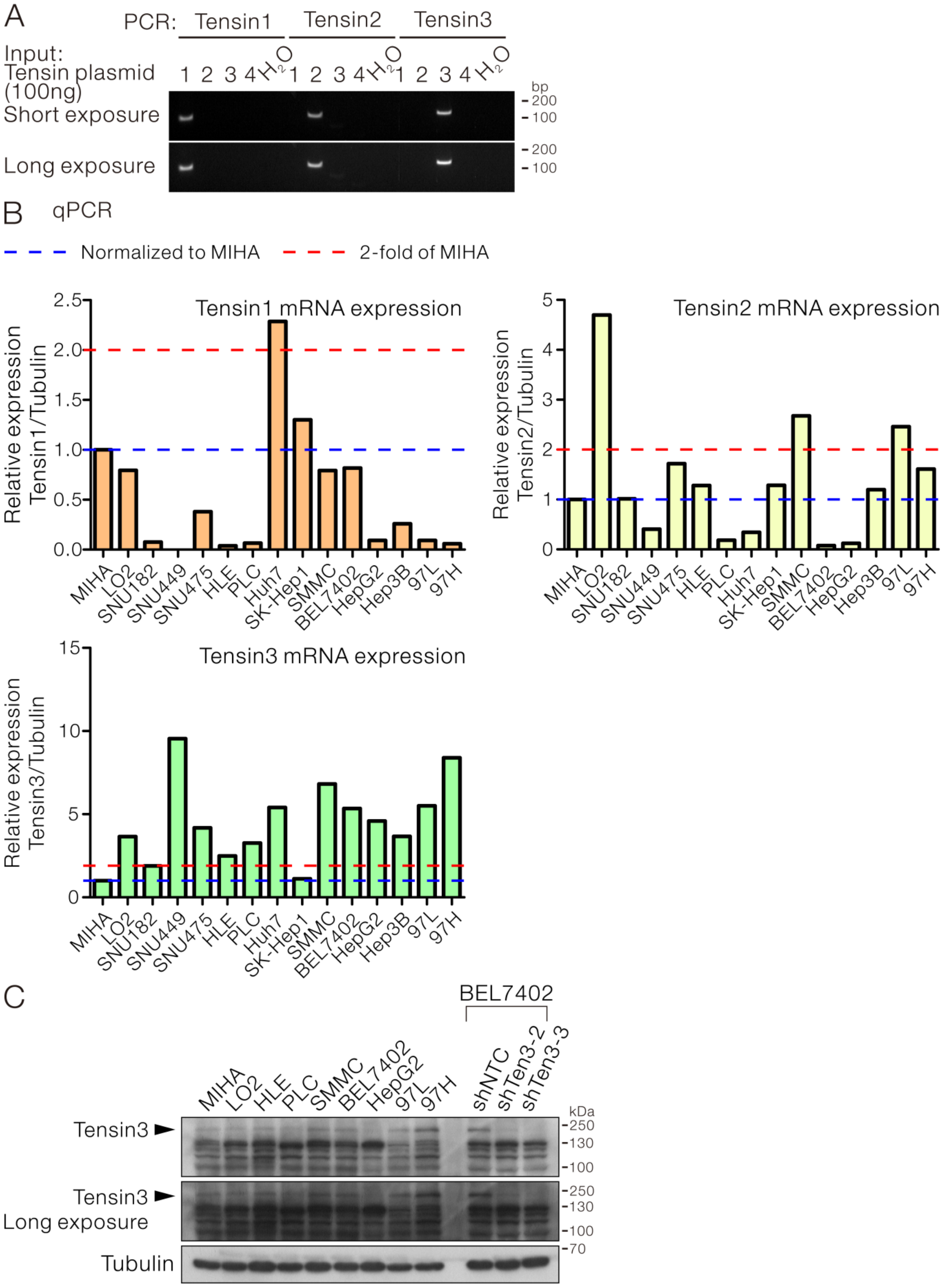
A
PCR: Tensin1 Tensin2 Tensin3
Input:
Tensin plasmid (100ng)
1 2 3 4 H2O 1 2 3 4 H2O 1 2 3 4 H2O
Short exposure
Long exposure
bp
200
100
B qPCR
Normalized to MIHA
2-fold of MIHA
Tensin1 mRNA expression
Relative expression Tensin1/Tubulin
Tensin2 mRNA expression
Relative expression Tensin2/Tubulin
Tensin3 mRNA expression
Relative expression Tensin3/Tubulin
MIHA LO2 SNU182 SNU449 SNU475 HLE PLC Huh7 SK-Hep1 SMMC BEL7402 HepG2 Hep3B 97L 97H
C
BEL7402
MIHA LO2 HLE PLC SMMC BEL7402 HepG2 97L 97H shNTC shTen3-2 shTen3-3
Tensin3
Tensin3
Long exposure
Tubulin
kDa
250
130
100
70

Supplementary Figure 1: Tensin1, -2 and -3 mRNA expression in HCC cells. A. Specificity of the Tensin1, -2 and -3 specific primers for qPCR assay. B. qPCR assay for Tensin1, -2 and -3 transcript expression in HCC cell lines. Tubulin served as the internal control. The normalized expression of each Tensin member was compared with the immortalized normal liver cell line MIHA. C. Western blotting for Tensin3 expression in HCC cells. Tubulin was used as the normalization control. The band position of the Tensin3 protein is indicated with the arrowhead. Cell lysates from the two Tensin3 stable knockdown BEL7402 cells (shTensin3-2 and -3) plus the non-targeted control (NTC) cells were included on the same blot as a control.

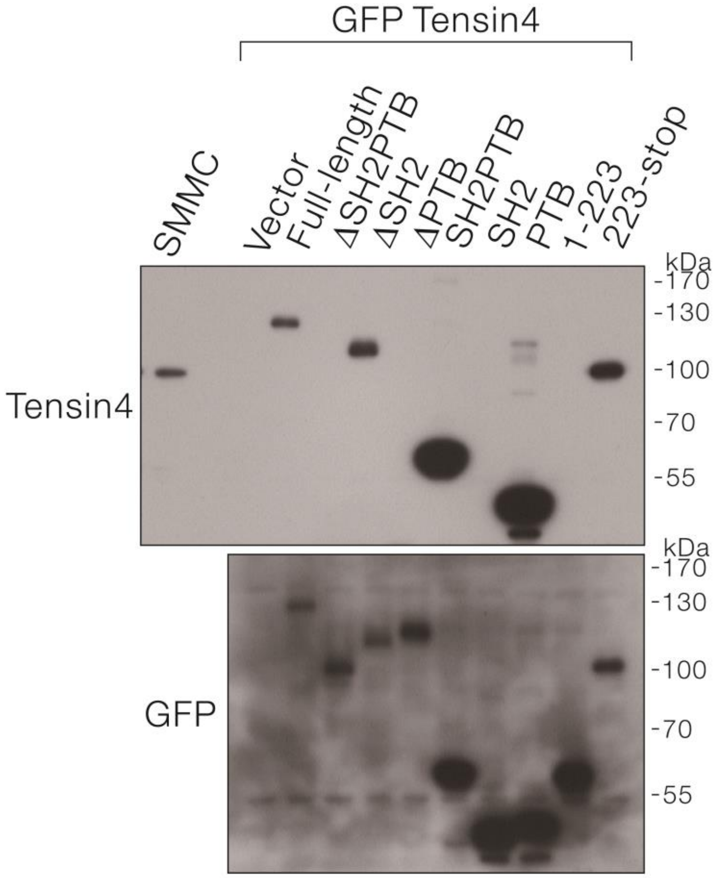

Supplementary Figure 2: Specificity of the Tensin4 antibodies used in the current study. Cell lysate from the parental SMMC-7721 cells (with relatively high Tensin4 expression) and HLE cells (with low Tensin4 expression) with the indicated GFP-Tensin4 constructs transiently transfected were subjected to Western blotting with Tensin4 antibodies. The successful transfection and expression of all Tensin4 constructs were confirmed by immunoblotting against GFP antibodies. The Tensin4 antibodies were able to detect Tensin4 expression in SMMC cells as well as Tensin4 wild type-transfected HLE cells. The antibodies recognized the Tensin4 PTB domain as epitope, supported by the observation that no detectable band was observed when Tensin4 mutants lacking the PTB domain (ΔSH2PTB, ΔPTB, SH2 and 1-223) were transfected.

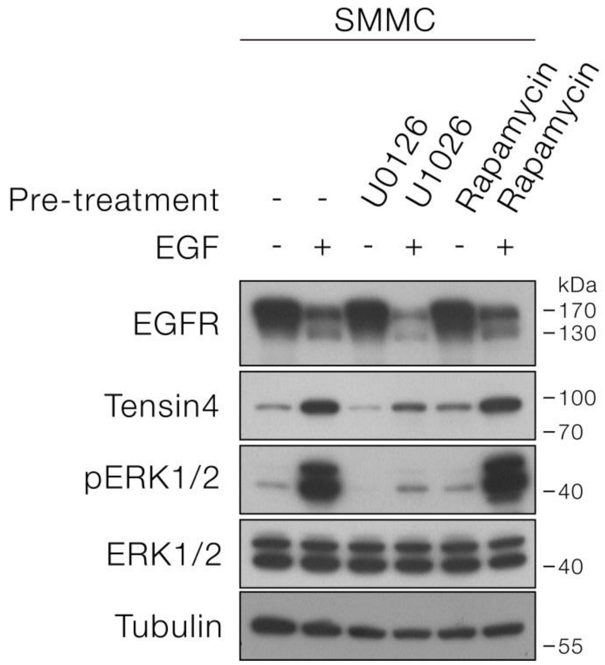

Supplementary Figure 3: Pre-treatment with of the mTORC1 inhibitor did not abolish the EGF-induced Tensin4 expression. SMMC-7721 cells were pre-treated with MEK inhibitor U0126 or mTORC1 inhibitor Rapamycin for 1 hour followed by the EGF treatment for 6 hours. Cell lysates were then collected and subjected to Western blotting for the indicated proteins.

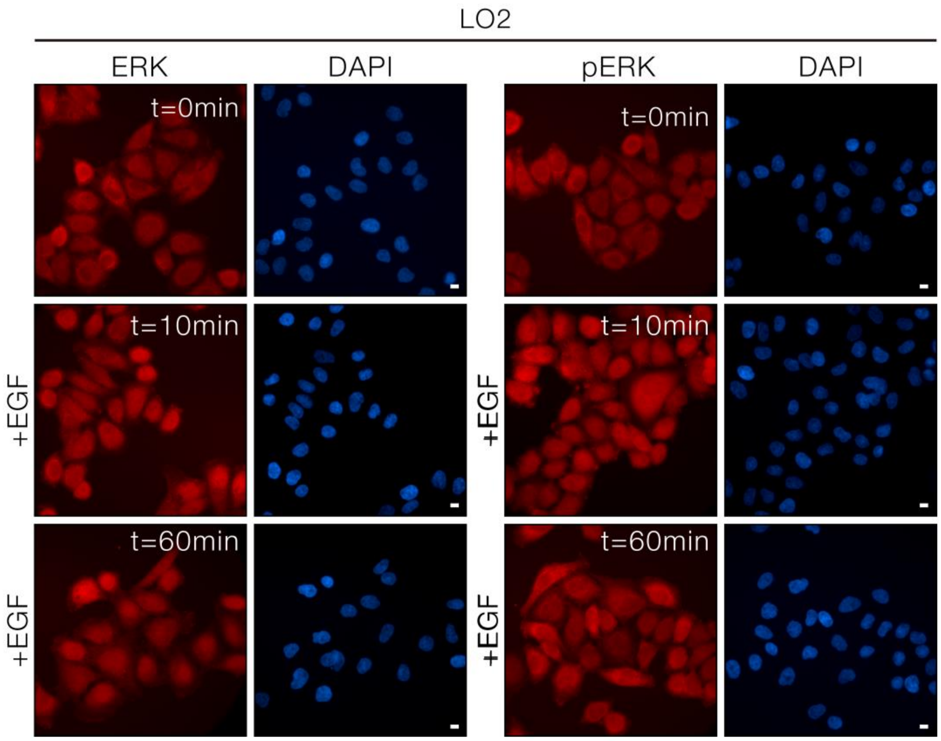

Supplementary Figure 4: EGF treatment induced nuclear translocation of ERK at specific time points. LO2 cells subjected to EGF induction for the indicated time points were fixed and examined by immunofluorescence for ERK1/2 and phospho-ERK1/2. The coverslips were counterstained with and DAPI for nuclei. Scale bar: 10 μm.

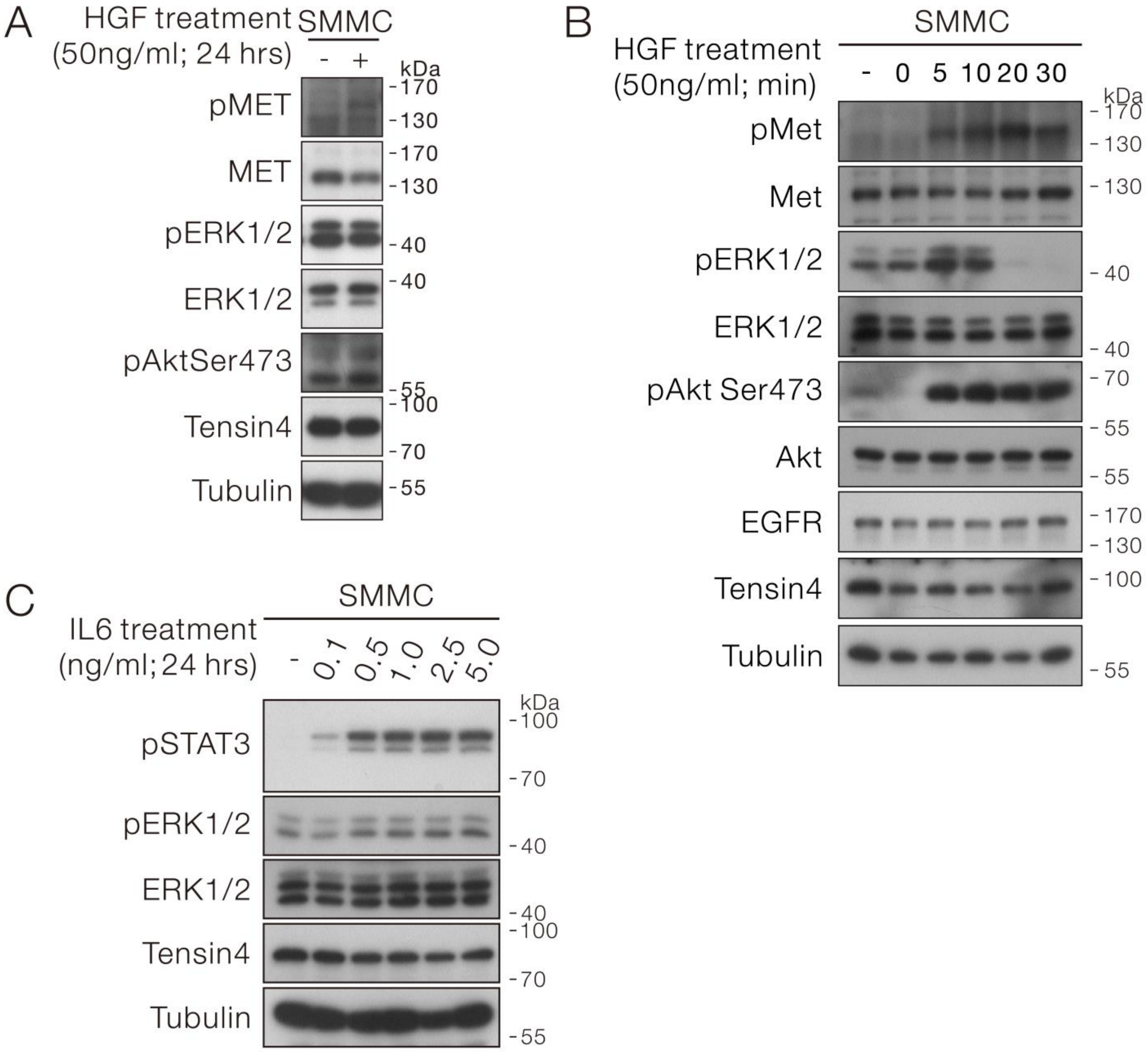

Supplementary Figure 5: HGF and IL6 treatment did not up-regulate Tensin4 expression in SMMC-7721 cells. A. SMMC-7721 cells were treated with 50ng/ml HGF for the indicated time points. Western blotting was performed to detect the activation of MET receptor, its downstream signaling activation and the Tensin4 expression level. B. SMMC-7721 cells were treated with IL6 for 24 hours at the indicated concentrations. Western blotting was performed to detect its downstream signaling activation and the Tensin4 expression level.

Supplementary Table 1. Clinicopathological data of the HCC patients with Tensin4 IHC analysis.

Characteristics	Sample cohort (n=30)
Sex	
Male	23 (76.7%)
Female	7 (23.3%)
Mean age (range)	52.9 (24-74)
Average tumor size (range, in cm)	6.5 (1-27)
Background liver disease	
Normal and chronic hepatitis	16 (53.3%)
Cirrhosis	14 (46.7%)
Liver invasion	
Absent	8 (26.7%)
Present	16 (53.3%)
Tumor microsatellite formation	
Absent	14 (46.7%)
Present	16 (53.3%)
Tumor encapsulation	
Absent	10 (33.3%)
Present	20 (66.7%)
Venous invasion	
Absent	18 (60%)
Present	12 (40%)
Cellular differentiation	
Edmondson grade I-II	3 (10%)
Edmondson grade III-IV	27 (90%)
TNM staging	
I-II	21 (70%)
III-IV	9 (30%)
